# Jaw Bone Density in Chronic Areca Nut Chewers and Nonchewers Using Digital Panoramic Radiography Indices as a Screening Tool for Osteoporosis: Protocol for a Comparative Evaluation

**DOI:** 10.2196/72041

**Published:** 2025-05-15

**Authors:** Aakanksha Tiwari, Suwarna Dangore-khasbage

**Affiliations:** 1 Department of Oral Medicine and Radiology Sharad Pawar Dental College and Hospital Datta Meghe Institute of Medical Sciences Wardha India

**Keywords:** areca nut, digital panoramic radiography, jaw bone density, osteoporosis, bone mineral density

## Abstract

**Background:**

Jaw bone density can be altered due to various factors including aging, bone pathologies, hormonal levels, medications affecting bone density, and undue stress posed by parafunctional and adverse habits. Of these factors, chronic areca nut chewing, which creates a heavy load on jaw bones, is a commonly encountered adverse habit in patients. Digital panoramic radiography (OPG) indices are an easy and cost-effective method to evaluate jaw bone density. Index values can also be used as a screening tool for osteoporosis.

**Objective:**

This study aims to compare and evaluate jaw bone density in chronic areca nut chewers and nonchewers using OPG indices as a screening tool for osteoporosis.

**Methods:**

Patients aged 20 years to 40 years reporting to the Department of Oral Medicine and Radiology with and without a history of chronic areca nut chewing will be recruited. OPG will be collected for all recruited patients. The mandibular cortical index, panoramic mandibular index, gonial index, antegonial index, antegoinal notch depth, and mental index will be calculated.

**Results:**

The values of these indices will be used to assess and compare osteoporosis in chronic areca nut chewers and nonchewers. Data will be entered and displayed in a tabular format, and correlations between osteoporosis and OPG index values will be determined. The study did not receive any external funding. Recruitment is expected to begin in summer 2025, and publication of the results is expected to occur in late 2026.

**Conclusions:**

Evaluation of jaw bone density in chronic areca nut chewers using OPG indices might prove to be a feasible and cost-effective technique for assessing osteoporotic bone changes. Although the gold standard modality is dual x-ray absorptiometry, its cost and availability create challenges for use in the general population. As osteoporosis of jaw bones does not usually present with symptoms, patients can be made aware of it using the findings from this evaluation. Hence, it will ultimately aid in early detection and prompt interventions, thereby halting disease progress.

**International Registered Report Identifier (IRRID):**

PRR1-10.2196/72041

## Introduction

Maxillary and mandibular jaw bones have different densities. Mandibular bone is more dense and compact than maxillary bone, which is cancellous bone. Alteration in bone density can occur due to aging, diseases affecting bone, and altered hormonal levels, especially in peri- and postmenopausal women. Osteoporosis, which affects bone density, is a common finding in women older than 40 years. It can also be seen in men with various diseases affecting the bones or taking medications that affect bone mineral density (BMD). The risk of osteoporosis increases with age and can have deleterious effect on the body. Early detection of this condition may help to halt progression and offer prompt treatment to the patients.

One of the reasons for altered jaw bone density is the amount and frequency of functional load (load posed on the mandibular bone due to continuous masticatory movement) to which it is subjected [1]. A continuous heavy masticatory load attributed to a continuous grinding action, such as when chewing areca nut, poses a risk of altered jaw bone density. This can be due to various reasons like bone remodeling in response to increased masticatory load and toxic elements leaching out of areca nut. Studies have been performed to evaluate jaw bone density in bruxers and nonbruxers, and the results revealed increased cortical thickening (ie, increased jaw bone density), which is a reaction to a continuous load applied to the bone [1].

India is a country where habits like areca nut chewing, use of tobacco in either smoked or smokeless forms, and alcohol consumption are extremely prevalent, especially among young adults. The effect of smoking on BMD has already been proven in many previous studies. Areca nut chewing increases the masticatory load on the jaw bones in the same manner as that seen in patients with bruxism. Furthermore, studies have revealed a decrease in vitamin D levels in chronic areca nut chewers, which in turn can affect the density of jaw bones [2]. This shows that areca nut chewing might alter the density of the jaw bones. Hence, the effect of areca nut chewing on bone density remains controversial.

Digital panoramic radiography (OPG) is a widely used imaging modality for primary screening in clinical practice. It has several significant advantages including feasibilty, ease of acquisition, being cost effective, a low radiation dose, and ability to depict the dentition as well as jaw bones together. Several indices studied on OPG are helpful for evaluating jaw bone density. The measurements are easy to perform and understand. Hence, the overall benefits of OPG make it a beneficial tool for evaluation of jaw bone density [3].

There is a paucity of studies related to jaw bone density in chronic areca nut chewers using OPG indices and evaluating the efficacy of OPG as a screening tool for osteoporosis [4].

Hence, the purpose of this study is to compare and evaluate jaw bone density in chronic areca nut chewers using panoramic radiographs and to detect the efficacy of the same as a screening tool for the detection of underlying undiagnosed osteoporosis.

## Methods

### Ethical Considerations

The study received approval from the Institutional Ethics Committee (IEC number DMIHER(DU)/IEC/2023/1203) of Datta Meghe Institute of Higher Education and Research, Sawangi (Meghe), Wardha.

Informed consent was obtained from all participants involved in the study. The data collected will not include any identifiable information. Patients will benefit from education on the adverse effects of areca nut chewing on jaw bone density and receive proper counseling to cease chewing. They will receive no other compensation.

### Participants

This prospective, observational, case control study will be conducted with patients aged 20 years to 40 years visiting the Department of Oral Medicine and Radiology with a chronic history of areca nut chewing. The patients will be divided into 2 groups: Group A (patients without a history of chronic areca nut chewing; control group) and Group B (patients with a chronic history of areca nut chewing; study group). The inclusion criteria are patients aged between 20 years and 40 years, dentulous patients with a chronic history of areca nut chewing, and dentulous patients with a complete set of dentition except the third molars. The exclusion criteria are patients with a history of smoking, alcohol consumption, or drug use; with any systemic disease affecting the bone; having undergone chemoradiotherapy for any reason; taking medications affecting bone density; with congenital anomalies, malignancy, or disorders of jaw bones; with a jaw bone fracture; with a history of a surgical intervention involving jaw bones; with a history of orthodontic treatment; with periodontal diseases; and with a history of bruxism.

### Sample Size

Participants will be divided into 2 equal groups. The sample size was calculated using N=B/C, where C=(E/S_Ϫ_)^2^, B=(Z_α +_ Z_β_)^2^, Z_α_ is the standard normal deviation for α (1.9600), and Z_β_ is the standard normal deviation for β (0.06745). C was 0.2500, and B was 6.9403, resulting in an N of 27.7614 for each group. Therefore, N was rounded up to the next highest integer to give a group size of 70.

### Methodology

Eligible patients reporting to the Department of Oral Medicine and Radiology and who are willing to participate in the study will be recruited. Each patient’s previous medical history will be collected to rule out systemic disease affecting jaw bone density, history of medications affecting the bone, and history of pathology affecting the jaw bones. Each patient’s previous dental history will be collected to rule out a history of orthodontic treatment or periodontal diseases. A detailed history of habits, including type, duration, and frequency, will be collected from each patient. A thorough clinical examination will be conducted to rule out missing teeth, root pieces, or impacted teeth. All patients will be undergo a panoramic radiographic examination using standard parameters.

**Figure 1:**
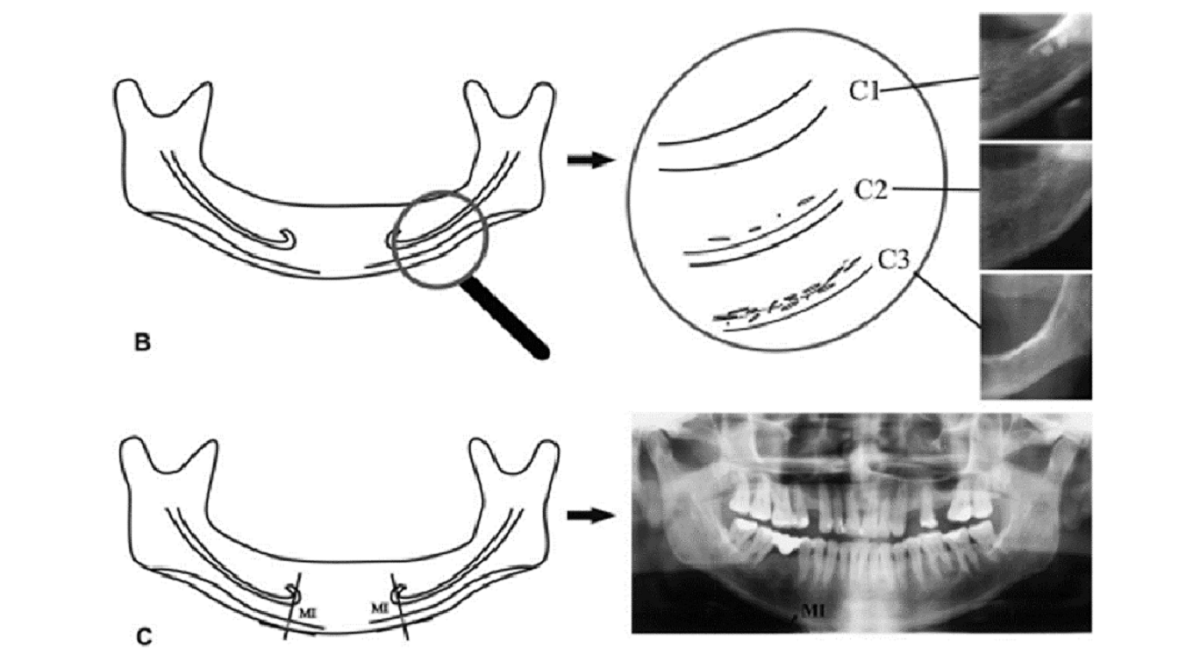
Graphic and radiographic representation for the calculation of the (B) mandibular cortical index and (C) mental index.

**Figure 2:**
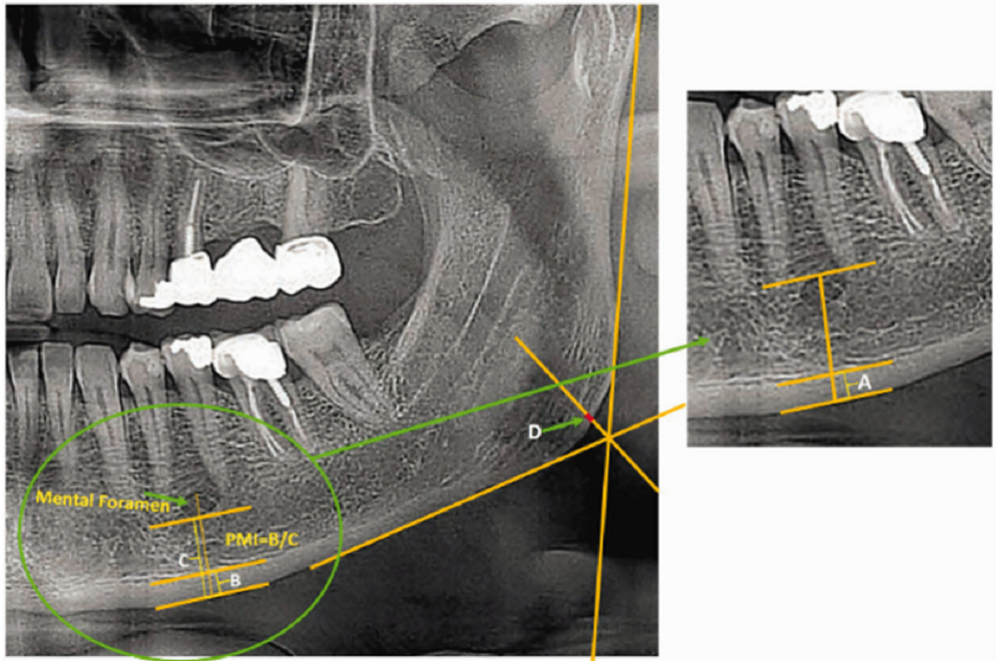
Graphic and radiographic representation for calculations of the panoramic mandibular index (width B/width C) and gonial index (width D).

**Figure 3:**
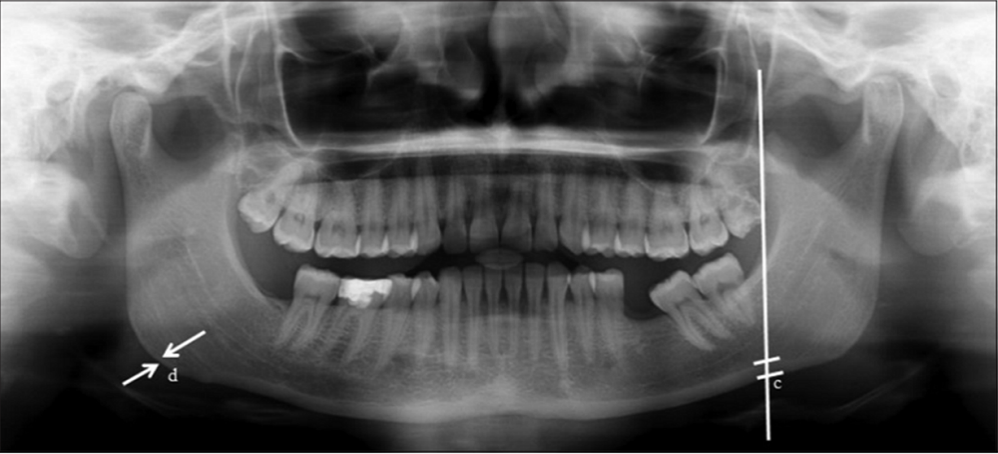
Radiographic representation of the determination of the antigonial index.

**Figure 4:**
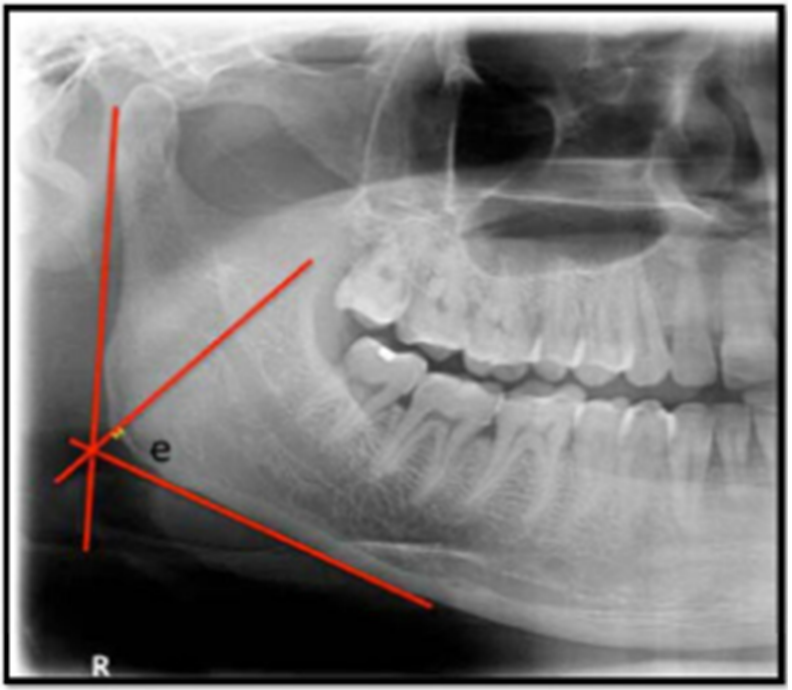
Graphic and radiographic representation for calculation of the gonial index: the length of “e” in mm.

**Figure 5:**
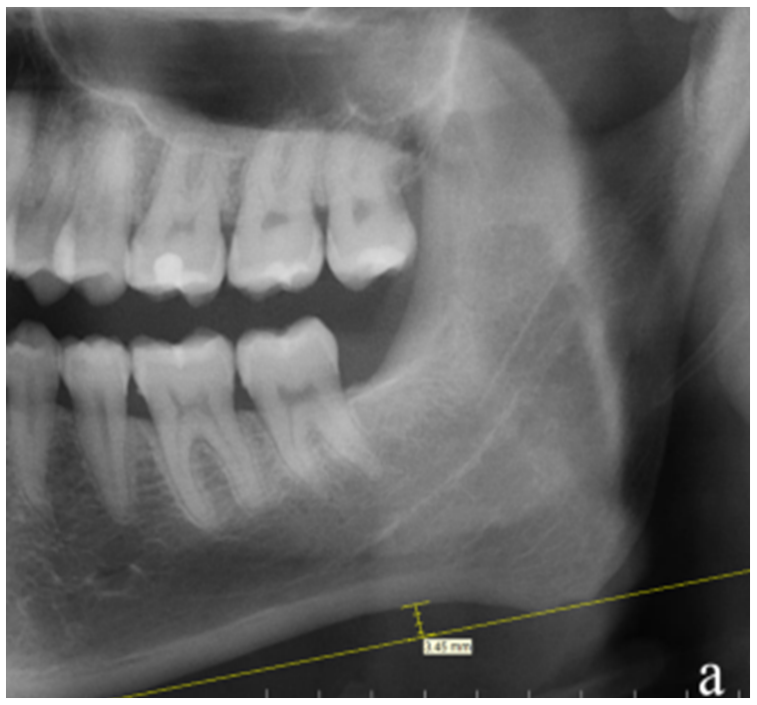
Graphic and radiographic representation for the calculation of the antegonial notch depth, which is calculated by measuring the depth of the notch from the line drawn tangent to the inferior border of the mandible.

Evaluation of jaw bone density will be based on the OPG indices as shown in Figures 1-5 [5,6]. The reference values for mandibular cortical index (MCI) and panoramic mandibular index (PMI) for normal, osteoporotic, and osteopenic bone that will be used are shown in Table 1 [7].

**Table 1:** Reference values for digital panoramic radiography indices to evaluate osteoporosis.

Bone density	Scores for the orthopatomogram indices	
	Mandibular cortical index	Panoramic mandibular index
Normal	4.56	0.33
Osteoporotic	3.39	0.24
Osteopenic	3.97	0.28

### Statistical Analysis

Reference values for MCI and PMI will be correlated with the OPG index values to determine the patients’ osteoporosis status.

## Results

The study did not receive any external funding. Recruitment is expected to begin in summer 2025, and results are expected to be published in late 2026.

The results from the analyses based on the values of OPG indices will be displayed in tabular form similar to that shown in Tables 2 and 3.

**Table 2:** Example representation of how the values of the digital panoramic radiography indices in the control group and study group will be reported.

Study group	Digital panoramic radiography indices					
	MCI^a^	PMI^b^	AI^c^	GI^d^	AND^e^	MI^f^
Control group	—^g^	—	—	—	—	—
Study group	—	—	—	—	—	—

^a^MCI: mandibular cortex index.

^b^PMI: panoramic mandibular index.

^c^AI: antegonial index.

^d^GI: gonial index.

^e^AND: antegonial notch depth.

^f^MI: mental index.

^g^Placeholder.

**Table 3:** Example representation of how bone quality based on the digital panoramic radiography indices in the control and study groups will be reported.

Study group	Calculated MCI^a^ value	Calculated PMI^b^ value	Status of bone after correlation of calculated values of MCI with reference values	Status of bone after correlation of calculated values of PMI with reference values
Control group	—^c^	—	—	—
Study group	—	—	—	—

^a^MCI: mandibular cortex index.

^b^PMI: panoramic mandibular index.

^c^Placeholder.

## Discussion

### Background

Chronic areca nut chewing is thought to decrease jaw bone density. Multiple reasons have been discussed, including remodeling of bone and toxic elements leaching out due to chronic areca nut chewing. A few studies, however, have suggested an increase in bone density due to chronic areca nut chewing. Hence, this study will aid with establishing more clarity over this dispute. As areca nut is a hard substance, the chronic masticatory actions associated with chewing areca nut should lead to increased jaw bone density; however, due to a decrease in vitamin D levels, there could be a negative impact on jaw bone density. As osteoporosis is asymptomatic, it is frequently overlooked and goes undiagnosed. Dual x-ray absorptiometry examinations are currently the gold standard for diagnosing osteoporosis based on BMD. However, “a large portion of the Indian elderly population cannot access this diagnostic modality due to its high cost and limited availability” [8]. In order to diagnose oral problems, dentists frequently use radiography in their daily work. The focus of recent studies has been on whether radiographic changes in the jaws might serve as a reliable, early osteoporosis diagnosis tool [8].

### Comparison With Prior Work

Bajoria et al [9] conducted a study to measure radiomorphometric indices in OPG to find interrelationships between the indices and age and sex. In the study, 23 patients were randomly selected to be examined. The results revealed that the MCI, PMI, mental index, and antigonial index can be effectively measured on OPG and could be used to determine osteoporosis. All indices were statistically significantly correlated with osteoporosis in both the younger and older age groups. All indices were lower in women than men [9]. Similarly, Suman and Redhu [10] conducted a study to assess relationships between BMD and radiomorphometric indices using panoramic radiography in tobacco users, and the authors concluded that tobacco had a detrimental effect on BMD as revealed by the MCI, PMI, and MI. Palaskar et al [11] conducted a study to assess relationships between personal habits like tobacco chewing, areca nut chewing, alcohol consumption, and BMD in Indian adult men aged 20 years to 60 years using OPG. They concluded that OPG indices, especially the antegonial index, were negatively affected in men with the personal adverse habits of interest. This radiography technique is readily available and reasonably priced. Dental practitioners might be the first to notice abnormalities in the mandible and make the necessary referrals because OPG is a standard diagnostic technique in the office [11].

Temur et al [12] evaluated the effect of anti-epileptic drugs (AEDs) on trabecular and mandibular cortical bone. The case group’s fractal dimension (FD) of the mandibular body and angle and mandibular cortical width were substantially lower than those of the control group (*P*<.001). Compared with second-generation medications, first- and third-generation AEDs were associated with reduced FD values in the ramus and angle (*P*≤.011). Users of first- and second-generation AEDs had lower mandibular FDs than users of third-generation drugs (*P*=.017). Although PMI increased with more than 1 year of AED use (*P*≤.02), drug use for at least 1 year was associated with considerably lower values for all FDs and mandibular cortical widths and more class 3 MCI assessments than short-duration use. Sex and age had no discernible impact [12]. A study was conducted by Önsüren and Temur [13] to examine the effects of likely sleep and awake bruxism on the mandibular trabecular bone structure in pediatric patients with bruxism using fractal analysis, OPG, and radiomorphometric measures. It concluded that bruxism can alter mandibular trabecular bone in children and teenagers. Just like in adult dentistry, fractal analysis can be used as a supplemental technique to identify the mandibular trabecular variations of patients with bruxism in pediatric dentistry [13]. Geçkil and Temur [7] investigated the effect of two different bisphosphonate types on bone using dental panoramic radiographs and compared these findings with those from a healthy cohort. This study demonstrated that changes in cortical bone structure are significantly influenced by the kind of bisphosphonate and the length of medication usage. It appears that bisphosphonates have a long-lasting effect on bone since these effects are persistent and unaffected by the amount of time that has passed since the last dose [7].

### Strengths

It is possible to screen using OPG indices because a history of smoking, drinking, and eating betel nut products may be regarded as a risk factor for the development of osteoporosis [14]. Evaluation of bone density in chronic betel nut chewers has not yet been assessed and compared with bone density in nonchewers. Although the literature includes some studies in which bone density was altered in areca nut chewers, the scarcity of the same leads to the need for further research in this field [5]. Hence, this study will aid with assessing bone density in chronic areca nut chewers and help with educating patients about the condition, thereby aiding with appropriate treatment approaches and halting the progression of the same.

### Limitations

The study will use OPG indices to evaluate jaw bone density; however, the indices cannot be compared with the gold standard dual x-ray absorptiometry to establish the efficacy of the OPG for determining jaw bone density due to unreliable patient compliance, high cost, and limited availability of dual x-ray absorptiometry.

### Conclusion

This study hypothesizes that smoking, areca nut, and alcohol consumption negatively impact mandibular bone density based on the existing literature, which suggests reductions in MCI, PMI, mental index, and antegonial index. Although a few studies have demonstrated increased bone density, the majority reflected a reduction. Dentists can detect these changes through routine panoramic radiography examinations and identify patients at risk for osteoporosis at an early stage. A reduction in jawbone density is a significant risk factor for implant placement, prosthetic planning, and periodontal treatments. Therefore, dental implant placement and prosthetic planning should be approached more cautiously in patients with decreased bone density.

## Abbreviations

AED: anti-epileptic drug
BMD: bone mineral density
FD: fractal dimension
MCI: mandibular cortical index
OPG: digital panoramic radiography
PMI: panoramic mandibular index
